# Crohn’s disease in endoscopic remission, obesity, and cases of high genetic risk demonstrates overlapping shifts in the colonic mucosal-luminal interface microbiome

**DOI:** 10.1186/s13073-022-01099-7

**Published:** 2022-08-15

**Authors:** Jonathan P. Jacobs, Maryam Goudarzi, Venu Lagishetty, Dalin Li, Tytus Mak, Maomeng Tong, Paul Ruegger, Talin Haritunians, Carol Landers, Philip Fleshner, Eric Vasiliauskas, Andrew Ippoliti, Gil Melmed, David Shih, Stephan Targan, James Borneman, Albert J. Fornace, Dermot P. B. McGovern, Jonathan Braun

**Affiliations:** 1 Vatche and Tamar Manoukian Division of Digestive Diseases, Department of Medicine, David Geffen School of Medicine at UCLA, Los Angeles, CA 90095-6949 USA; 2 Division of Gastroenterology, Hepatology and Parenteral Nutrition, Veterans Administration Greater Los Angeles Healthcare System, Los Angeles, USA; 3 Lerner Research Institute, Cleveland Clinic, Cleveland, USA; 4 F. Widjaja Foundation Inflammatory Bowel and Immunobiology Research Institute, Cedars-Sinai Medical Center, Los Angeles, USA; 5 National Institute of Standards and Technology, Gaithersburg, USA; 6 Department of Pathology and Laboratory Medicine, David Geffen School of Medicine at UCLA, Los Angeles, USA; 7 Department of Plant Pathology and Microbiology, University of California Riverside, Riverside, USA; 8 Department of Medicine, Keck School of Medicine of USC, Los Angeles, USA; 9 Department of Biochemistry and Molecular & Cellular Biology, Georgetown University, Washington, USA

**Keywords:** Crohn’s disease, Obesity, Microbiome, Mucosal-luminal interface, Disease behavior, Disease progression, Genetic risk score

## Abstract

**Background:**

Crohn’s disease (CD) patients demonstrate distinct intestinal microbial compositions and metabolic characteristics compared to unaffected controls. However, the impact of inflammation and underlying genetic risk on these microbial profiles and their relationship to disease phenotype are unclear. We used lavage sampling to characterize the colonic mucosal-luminal interface (MLI) microbiome of CD patients in endoscopic remission and unaffected controls relative to obesity, disease genetics, and phenotype.

**Methods:**

Cecum and sigmoid colon were sampled from 110 non-CD controls undergoing screening colonoscopy who were stratified by body mass index and 88 CD patients in endoscopic remission (396 total samples). CD polygenic risk score (GRS) was calculated using 186 known CD variants. MLI pellets were analyzed by 16S ribosomal RNA gene sequencing, and supernatants by untargeted liquid chromatography-mass spectrometry.

**Results:**

CD and obesity were each associated with decreased cecal and sigmoid MLI bacterial diversity and distinct bacterial composition compared to controls, including expansion of *Escherichia/Shigella*. Cecal and sigmoid dysbiosis indices for CD were significantly greater in obese controls than non-overweight controls. CD, but not obesity, was characterized by altered biogeographic relationship between the sigmoid and cecum. GRS was associated with select taxonomic shifts that overlapped with changes seen in CD compared to controls including *Fusobacterium* enrichment. Stricturing or penetrating Crohn’s disease behavior was characterized by lower MLI bacterial diversity and altered composition, including reduced *Faecalibacterium*, compared to uncomplicated CD. Taxonomic profiles including reduced *Parasutterella* were associated with clinical disease progression over a mean follow-up of 3.7 years. Random forest classifiers using MLI bacterial abundances could distinguish disease state (area under the curve (AUC) 0.93), stricturing or penetrating Crohn’s disease behavior (AUC 0.82), and future clinical disease progression (AUC 0.74). CD patients showed alterations in the MLI metabolome including increased cholate:deoxycholate ratio compared to controls.

**Conclusions:**

Obesity, CD in endoscopic remission, and high CD genetic risk have overlapping colonic mucosal-luminal interface (MLI) microbiome features, suggesting a shared microbiome contribution to CD and obesity which may be influenced by genetic factors. Microbial profiling during endoscopic remission predicted Crohn’s disease behavior and progression, supporting that MLI sampling could offer unique insight into CD pathogenesis and provide novel prognostic biomarkers.

**Supplementary Information:**

The online version contains supplementary material available at 10.1186/s13073-022-01099-7.

## Background

Crohn’s disease (CD) is a chronic inflammatory bowel disease (IBD) of the digestive tract with substantial morbidity and mortality due to intestinal ulceration, obstruction, and perforation leading to malnutrition, infections, and debilitating symptoms [1]. Prevalence is substantial (~0.3%) but stabilizing in the US and other industrialized countries, while incidence is rising globally across newly westernizing countries [2]. The pathophysiology of CD involves a combination of genetic susceptibility (concordance in twins of 30–50%, and over 200 genetic loci of genome-wide significance [3]) and environmental risk factors [2, 4]. The intestinal microbiome was initially implicated in disease development based on animal models (e.g., [5–7]). When extended to studies of human microbiome (see foundational studies [8–16]; reviewed in [17]), CD patients compared to healthy populations are distinguished by lower microbial diversity, expansion, and (more notably) depletion of >30 microbial taxa, temporal instability [18–20], and altered production of many classes of metabolites and proteins with emerging documentation of their ecologic and host bioactivity [21–23]. These findings strongly support the hypothesis that CD involves a perturbation of the microbiome, termed dysbiosis, that synergizes with environmental and genetic risk factors to incite intestinal inflammation [24, 25].

Existing studies have provided insight into the link between the microbiome and CD but many questions remain. First, while both host genetics and gut microbiome composition are associated with CD, genome-wide analysis has uncovered very limited impact of host genetics on microbial composition [26–29]. This has led to the view that mechanisms of CD genetic risk act in parallel rather than upstream of microbiome disease traits. However, the advent of genome-wide CD polygenic risk score methodologies (GRS) offers a novel and more statistically powerful method to reconsider the relationship of host genetics to disease-associated traits such as microbiome composition, by testing them in healthy populations independent of CD disease state [30–32]. Second, CD is heterogeneous in phenotype, including diverse location patterns as well as clinically distinct categories of disease behavior summarized under the Montreal classification as stricturing (B2), internal penetrating (B3), and non-stricturing/non-penetrating (B1) [33]. The contributions of the microbiome to disease behavior have received limited attention to date [20, 34, 35]. Third, most studies of healthy control populations have used feces to characterize the microbiome. The mucosal microbiome of non-IBD individuals and its relation to other microbiome-associated conditions such as obesity and metabolic disorders [36, 37] or to genetic factors remain largely uncharacterized. This is an important limitation as studies comparing fecal and mucosal samples have found that the fecal microbiome is not representative of the mucosal microbiome, and more robust microbial differences have been found in the tissue microbiome than the fecal microbiome when comparing CD to controls [11, 38]. Fourth, existing moderate- to large-sized studies of the CD microbiome have included patients with active disease or defined remission by clinical parameters, which have been shown in CD to correlate poorly with inflammation measured by biomarkers and endoscopic appearance [11–14, 39, 40]. As inflammation itself appears to alter the gut microbiome as seen in animal models and in human studies associating severity of inflammation and therapeutic response to microbial profiles, it is unclear to what extent the reported dysbiosis associated with CD truly reflects an underlying disease association as opposed to microbial markers of ongoing intestinal inflammation [11, 12, 41].

We undertook a study to address these gaps in our understanding of the colonic mucosal microbiome of CD in remission. In deciding which specimen types to include, we focused on the mucosal-luminal interface (MLI). This represents colonic mucus and adherent microbes present after bowel preparation which can be released by lavage of the colonic surface during colonoscopy [42]. This provides insight into microbes in close proximity to the mucosa that have been shown to be distinct from those in the lumen and that may be most biologically pertinent for regulating host mucosal phenotype. A study comparing colonic lavage samples and colonic biopsies found that they had roughly comparable microbial profiles [43]. Unlike biopsies, which predominantly contain human tissue, MLI sampling yields a mix of bacterial and human material that we and others have shown is amenable for metabolomics, proteomics, shotgun metagenomics, and viromics [42, 44–49]. MLI proteomics, in particular, has been demonstrated to yield panels of proteins that can differentiate IBD from non-IBD as well as IBD phenotypes [45, 47, 48]. Microbiome sequencing of MLI samples has been utilized to identify microbial associations with type 2 diabetes, systemic sclerosis, and colorectal cancer [50–52].

In this study, we performed cross-sectional sampling of the MLI microbiome of CD patients in endoscopic remission and non-IBD controls, stratified by obesity status. This allowed for assessment of mucosal microbiome profiles in obesity and adjustment for the confounding effects of obesity in comparisons of CD to controls.

## Methods

### Cohort recruitment and sample collection

Eighty-eight CD patients undergoing colonoscopy and 110 controls without IBD undergoing screening colonoscopy were recruited from endoscopy suites at Cedars-Sinai Medical Center between 6/29/2011 and 3/19/2014. The demographic details of this cohort are summarized in Table 1. Details of cohort recruitment strategy and sampling methodology were previously reported [42, 44, 45]. For convenience, we highlight the following details. All CD patients were reported by their gastroenterologists to be in clinical remission at the time of collection. The participating study endoscopists, who were all experienced IBD specialists, confirmed that study patients were in endoscopic remission at the time of sampling based upon the absence of visible signs of active inflammation such as linear or aphthous ulceration or cobblestoning. Controls undergoing screening colonoscopy were validated by normal endoscopic appearance of the colon during colonoscopy. The subjects underwent lavage of the sigmoid colon and cecum at sites without visible pooled luminal content with 30 mL of sterile 0.9% saline passed through the endoscope channel. The lavaged content was aspirated by vacuum suction into a collection trap, typically yielding over 20 mL, then immediately transferred to ice. Within the same day, the lavaged samples were centrifuged at 4000*g* for 30 min to separate the sample into a pellet and supernatant, which were stored at −80°C until future microbiome and metabolomics analysis, respectively. A clinical research coordinator collected relevant metadata including age, gender, body mass index (BMI), and Montreal classification (disease behavior and disease location). Clinical chart review for longitudinal outcomes was performed in October 2017. Peripheral blood was collected from all patients for genetic analysis. The Cedars-Sinai Medical Center Institutional Review Board approved this research and the protocols governing participants (IRB #3358). All subjects provided informed consent to participate. The following datasets were generated for this study: 16S rRNA gene sequencing (CD—88 sigmoid lavage samples, 88 cecum lavage samples; non-IBD—110 sigmoid lavage samples, 109 cecum lavage samples), global untargeted metabolomics (CD—87 sigmoid lavage samples, 86 cecum lavage samples; non-IBD—108 sigmoid lavage samples, 105 cecum lavage samples), and genetic risk score (75 CD blood samples and 97 non-IBD blood samples). Details of data production are provided in the “Methods” subsections below.

**Table 1:** Cohort demographics. Continuous variables are shown as median (interquartile range). Significance of demographic data was determined by Fisher’s exact test for categorical data and the Wilcoxon rank-sum test for continuous data

	CD (***n***=88)	Control (***n***=110)	***p***-value
Gender			
Male	47%	70%	0.001
Female	53%	30%	
Race/ethnicity			
Caucasian	91%	95%	0.78
Hispanic	3%	0%	
African-American	2%	1%	
Asian/Pacific Islander	3%	2%	
Non-white Hispanic	3%	3%	
Age	41 (29–53)	64 (57–73)	2 × 10^−16^
BMI	23.5 (21.0–26.2)	25.8 (23.6–27.8)	0.0001
GRS	0.35 (−0.22–1.0)	−0.07 (−0.80–0.68)	0.003
Age at diagnosis	24 (19–33)		
Duration (years)	11 (6–17)		
CD location			
L1 = Ileal	15%		
L2 = Colonic	11%		
L3 = Ileocolonic	74%		
Upper GI involvement	8%		
Perianal disease	26%		
CD behavior			
B1 = Non-stricturing	34%		
B2 = Stricturing	32%		
B3 = Penetrating	34%		
Medication			
5-aminosalicylate	47%		
Immunomodulator	29%		
Biologic (anti-TNF)	57%		

### Genetic risk score

DNA was extracted from peripheral blood and applied to Immunochip, a custom platform containing nearly 200,000 single-nucleotide polymorphisms (SNPs) near genes related to immune function and inflammatory disease [30]. Quality control for genotype data was performed as previously described [30]. Gene risk scores (GRS) were calculated using the weighted sum of 186 SNPs associated with CD, including SNPs that are also associated with ulcerative colitis [30–32]. The SNPs were assumed to be independently associated with risk. For each SNP, an additive genetic model was calculated then the log odds ratio was multiplied by the number of corresponding risk alleles (0, 1, or 2). The GRS was calculated by summing these products across all genes according to the following formula: GRS = ∑_*i*_*β*_*i*_*G*_*i*_.

### 16S rRNA gene sequencing

Genomic DNA was extracted from the 395 pelleted samples (a single cecal lavage pellet was lost during handling) using the PowerSoil DNA Isolation Kit (MO BIO Laboratories, Carlsbad, CA, USA) with a 30-s beat-beating step in a Mini-Beadbeater-16 (BioSpec Products, Bartlesville, OK, USA) [53]. Polymerase chain reaction amplification of bacterial 16S rRNA genes was performed using PCR primers (F515/R806) targeting the V4 hypervariable region, with the reverse primers including a Golay barcode [54]. PCR products were purified using the MinElute 96 UF PCR Purification Kit (Qiagen, Valencia, CA, USA). DNA sequencing (100 bp reads) was performed using an Illumina HiSeq 2000 (Illumina, Inc., San Diego, CA, USA) as previously described [55]. Raw sequence data was demultiplexed and filtered in QIIME v1.9.1 using split_libraries_fastq with q=19 [56]. Deblur v1.1.0 was used with default parameters and min-reads 10 to denoise the data into amplicon sequence variants (ASVs) [57]. Taxonomy was assigned using the RDP classifier implemented in the assignTaxonomy function of the R package dada2 v1.16.0 and the Silva v138 database [58, 59]. Three samples with fewer than 50,000 sequences were excluded from the analysis. The sequence depth of the remaining samples ranged from 50,862 to 821,276, with a mean depth of 411,244.

### Microbiome data analysis

Alpha diversity was assessed using Chao1 and Shannon index with data rarefied to 50,862 sequences using the estimate_richness function of Phyloseq v1.32.0 in R v4.0.2 [60]. Statistical significance was assessed using multivariate ANOVA with post hoc Tukey implemented with the aov and TukeyHSD functions in R v4.0.2. Beta diversity analysis was performed with the vegdist function of the R package vegan v2.5-6 using Bray-Curtis dissimilarity [61]. These results were visualized by principal coordinates analysis (pcoa function in the R package ape v5-4.1); ellipses representing 95% confidence intervals were added using the stat_ellipse function of ggplot2 v3.3.5 in R. Permutational multivariate analysis of variance using distance matrices was performed with the adonis function in vegan v2.5-6 with 100,000 permutations to determine statistical significance of differences in beta diversity [62].

Differential abundance testing was performed using non-rarefied 16S rRNA sequence data filtered to remove ASVs present in less than 25% of samples. The resulting filtered datasets were analyzed using DESeq2 v1.28.1 implemented through Phyloseq v1.32.0 in R [60, 63]. This algorithm performs normalization using size factors estimated by the median-of-ratios method, employs an empirical Bayesian approach to shrink dispersion, and fits the data to multivariate negative binomial models [64]. *p*-values for variables in the linear models (e.g., IBD status) were converted to *q*-values using qvalue v2.20.0 in R v4.0.2 to correct for multiple hypothesis testing [65]. ASVs with *q*-values below 0.05 or 0.1 (for GRS analyses) and mean normalized relative abundance > 10^−5^ were considered significant. Similar analyses were also performed with the 16S rRNA sequence data summarized at the phylum level. Dysbiosis indices represent the log of the sum of relative abundances of taxa significantly enriched in CD by DESeq2 models divided by the sum of relative abundances of taxa depleted in CD.

To assess functional capacity of the microbiome for bile acid metabolism, the metagenome was predicted as relative abundances of KEGG orthologies using the PICRUSt2 function of QIIME2 v2019.10 [66]. Bacterial genes involved in bile acid 7α-dehydroxylation were identified based on the MetaCyc annotation for this pathway and matched to the corresponding KEGG orthologies: *baiB* (K15868), *baiA* (K15869), *baiCD* (K15870), *baiF* (K15871), *baiE* (K15872), and *baiN* (K07007) [67]. Relative abundances of these genes were added to generate an overall relative abundance for the bile acid 7α-dehydroxylation pathway.

### Ultra-performance liquid chromatography-mass spectrometry (LC-MS) untargeted metabolomics

Frozen aliquots of MLI supernatant from 386 samples underwent untargeted metabolomics analysis. Oasis MCX solid-phase extraction sorbents (Waters) were used to remove any potential interfering clinical materials such as polyethylene glycol. The aliquots were then sonicated at 37 °C for 90 s, chilled on ice, mixed in 150 μL of Optima acetonitrile containing internal standards, 4-nitrobenzoic acid and debrisoquine, then centrifuged. Supernatant was placed in a new tube, dried under a gentle stream of N_2_, and resuspended in solvent A (98% water, 2% acetonitrile, and 0.1% formic acid) for LC-MS. The MS analysis was performed by injecting 5 μL of each sample into a reverse-phase 50 × 2.1 mm H-class ultra-performance liquid chromatography Acquity 1.7-μM BEH C18 column (Waters) coupled to a time-of-flight mass spectrometry. The mobile phase consisted of solvent A and 100% acetonitrile containing 0.1% formic acid (solvent B). The Premier Q-TOF Xevo G2-S mass spectrometer (Waters) was operated in the positive (ESI+) and negative (ESI−) electrospray ionization modes scanning a 50–1200 m/z range. The following 13-min gradient was used: 95%/5% solvent A/solvent B at 0.45 ml/min for 8 min, 50%/50% solvent A/solvent B for 2 min, 2%/98% solvent A/solvent B for 2 min, and 95%/5% solvent A/solvent B for the remaining 1 min. The lock-spray consisted of leucine-enkephalin (556.2771 [M+H]^+^ and 554.2615 [M−H]^−^). The MS data were acquired in centroid mode and processed using MassLynx software (Waters Corp, Milford, MA, USA) to construct a data matrix consisting of the retention time, *m/z*, and intensity (via the peak area normalized to protein concentration) for each ion. A total of 4441 ions were detected in the two acquisition modes. Our in-house statistical analysis program was used to putatively identify ions, utilizing the Human Metabolome Database (HMDB), LipidMaps, the Kyoto Encyclopedia of Genes and Genomes (KEGG) database, and BioCyc allowing for the following adducts within a 20 parts per million (ppm) mass error window: H+, Na+, and/or NH4+ in the ESI+ mode; H− and Cl− in the ESI− mode [68].

### Metabolomics data analysis

Raw peak intensities for spectral features present in greater than 10% of samples underwent KNN imputation in R with *k*=10 using an R script provided with a publication [69]. The resulting imputed datasets underwent further analysis using the MetaboAnalyst R package v3.0.3 [70]. Filtering was performed by the “iqr” (interquartile range) criteria, data was log-transformed, and data underwent quantile normalization. Batch effect correction was performed using Combat as implemented in the PerformBatchCorrection function of MetaboAnalyst v3.0.3 [71]. Data was visualized by principal coordinates analysis of Euclidean distances. Differential abundance of spectral features was assessed by multivariate general linear models implemented in the limma package v3.44.3 in R v4.0.2 incorporating gender and obesity. *p*-values obtained from limma were process using the mummichog function of MetaboAnalyst v3.0.3 for identification of metabolic pathways enriched in differentially abundant features [72]. Pathway enrichment *p*-values were estimated using 100 permutations with gamma modeling of the permutations. Microbe-metabolite correlations were calculated with the cor function in R v.4.0.2 using Spearman’s correlation of residuals from multivariate models implemented in limma (metabolites) and DESeq2 (microbes).

### Random forests classifiers

Microbiome and metabolomics datasets were split 60%/40% into training and test subsets, respectively, using the createDataPartition function of the caret package v6.0-88 in R v4.0.2 [73]. Random forests classifiers were created using the train function of caret with mtry=2 and 1001 trees [74]. Features were inputted into the algorithm if they were significantly associated with the trait of interest in multivariate models. An initial classifier was created then all features with an importance score greater than 2 in the preliminary classifier were used to construct a refined classifier with fewer features. The accuracy of the final random forests classifiers was assessed using the test subset of the data with confidence intervals determined by bootstrapping. This was performed with the roc.test function of the pROC package v1.16.2 in R [75].

## Results

### Obesity is associated with reduced bacterial diversity and pathobiont expansion in the MLI microbiome

**Fig. 1:**
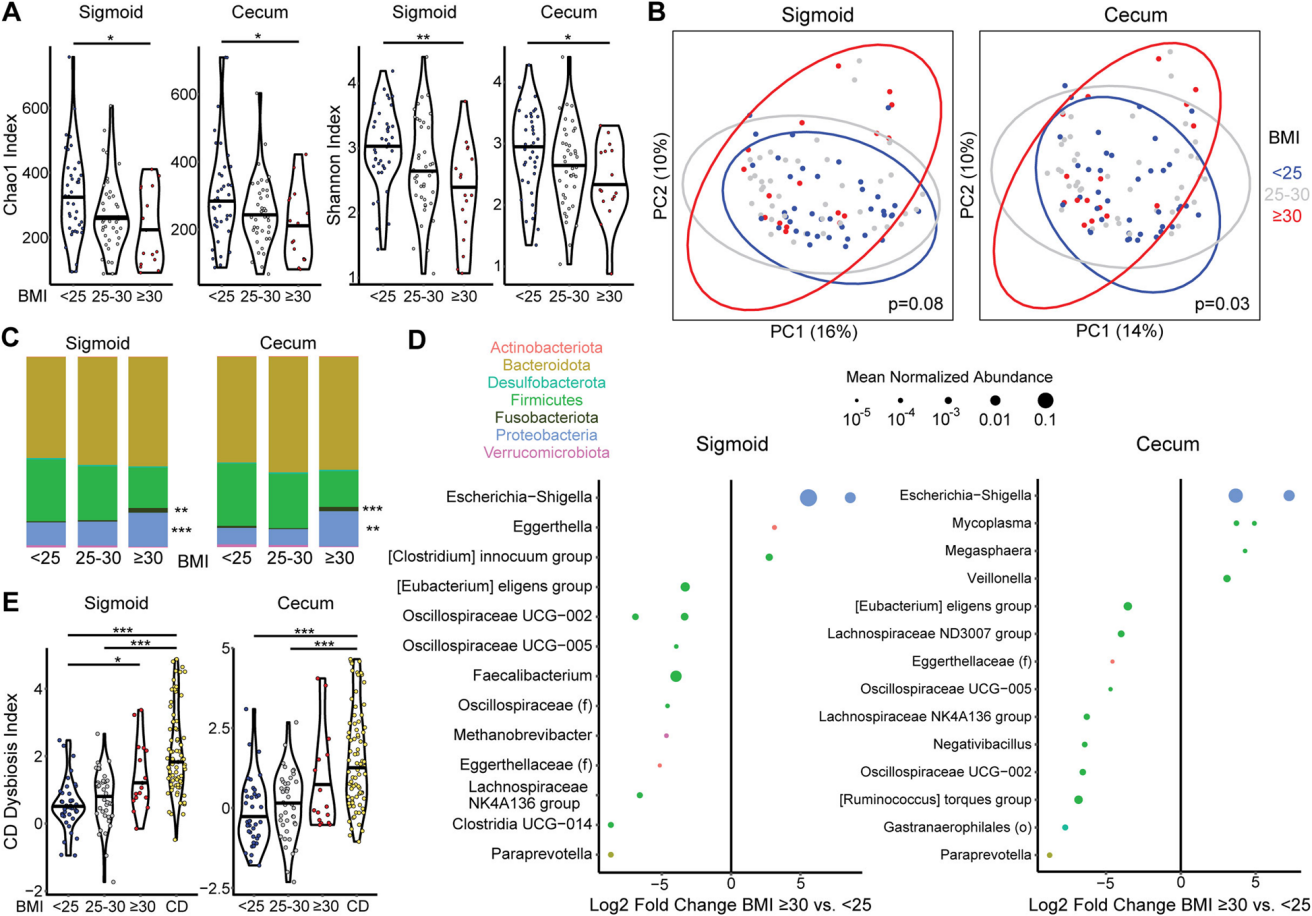
Obesity disrupts the mucosal-luminal interface (MLI) microbiome of non-IBD controls with shifts that parallel those in Crohn’s disease (CD). **A** Alpha diversity metrics (Chao1 and Shannon indices) are shown for the sigmoid and cecum MLI microbiome of non-IBD controls subdivided into three BMI categories. Significance was determined by ANOVA adjusting for gender and age with post hoc Tukey. *p<0.05, **p<0.01. **B** Principal coordinates analysis (PCoA) plot based on Bray-Curtis dissimilarity visualizing the cecal and sigmoid microbiome of controls subdivided into three BMI categories (indicated by color). Each dot represents one subject; ellipses represent 95% confidence intervals. *p*-values calculated by multivariate Adonis adjusting for gender and age. **C** Taxonomic summary at the phylum level of the cecal and sigmoid colon MLI microbiome of controls. *q<0.05, **q<0.01, ***q<10^−4^ for obese vs. BMI <25 in DESeq2 models adjusting for gender. **D** Differential ASVs in obese vs. normal weight controls in the cecal MLI microbiome from DESeq2 models controlling for gender and age. Effect size is represented as the log2 fold change between CD and control. Size of each dot is proportional to normalized relative abundance. Only ASVs with a mean normalized relative abundance greater than 10^−5^ are included. Color corresponds to phylum. ASVs are grouped into genera; those without an assigned genus are represented as unclassified members of families (f) or orders (o). **E** CD dysbiosis index, representing the log ratio of taxa enriched in CD to taxa depleted in CD, is shown for CD patients and controls stratified into three BMI categories. Significance determined by Kruskal Wallis with post hoc Dunn test corrected by Benjamini-Hochberg. *p<0.05, ***p<0.001

Individuals without IBD undergoing screening colonoscopy were recruited as a control group for this study. The majority were overweight or obese, presenting an opportunity to investigate mucosal microbiome profiles of obesity to build on the existing literature on fecal microbiome alterations and investigate whether obesity is a confounding factor in CD vs. non-IBD comparisons [36]. Obesity status was represented by BMI categories based on cutoffs of 25 and 30 for overweight and obese, respectively. This approach was taken to increase applicability to the CD cohort, which includes both overweight/obese subjects as well as a significant subset who are underweight as a consequence of disease. This would render invalid any linear relationships of the microbiome with BMI derived from an overweight population control cohort. Bacterial diversity was found to be significantly reduced in the sigmoid and cecum of obese non-IBD subjects compared to those with BMI<25 by metrics of richness alone (Chao1 index) and richness combined with evenness (Shannon index) (Fig. 1A). Overweight subjects (BMI 25–30) had intermediate bacterial diversity that did not significantly differ from either the obese or the BMI<25 groups. Differences in bacterial composition across samples were assessed by Bray-Curtis dissimilarity and visualized by principal coordinate analysis (PCoA) (Fig. 1B). Obesity category (BMI<25, BMI 25–30, or BMI>30) was significantly associated with variation in Bray-Curtis dissimilarity across non-IBD samples by adonis after adjusting for gender and age in the cecum but did not reach significance in the sigmoid. Analysis of only subjects with BMI>30 vs. those with BMI<25 demonstrated significant associations of obesity category with microbial composition at both sites (*p*=0.007 in the cecum, *p*=0.005 in the sigmoid). At the phylum level, obese subjects had increased abundance of Proteobacteria and Fusobacteriota in both the sigmoid and cecum as compared with subjects with BMI <25 (Fig. 1C). Differentially abundant amplicon sequence variants (ASVs) were identified using DESeq2, an algorithm that employs an empirical Bayesian approach to shrink dispersion and fits the data to negative binomial models. Obesity as compared with BMI<25 was associated with marked expansion of a highly abundant *Escherischia-Shigella* ASV in both the sigmoid and cecal MLI as well as expansion of *Mycoplasma* ASVs in the cecum, with depletion of ASVs largely belonging to genera within the Firmicutes phylum such as *Faecalibacterium* (Fig. 1D).

### The MLI microbiome of CD patients in endoscopic remission is characterized by lower bacterial diversity, pathobiont enrichment, and depletion of anti-inflammatory microbes

All CD subjects included in this study were confirmed to be in endoscopic remission at the time of sample collection. There were equal numbers of each of the three disease behaviors: 34% with non-stricturing, non-penetrating CD (B1), 32% with stricturing disease (B2), and 34% with internal penetrating (B3) disease (Table 1). Most CD subjects (74%) had ileocolonic (L2) disease with 15% having ileal (L1) and the remaining 11% having colonic (L3) disease. The majority were on treatment with a biologic (57%; all of these were receiving an anti-TNF agent including infliximab, adalimumab, or certolizumab), with 47% of patients receiving 5-aminosalicylates (47%) and 29% an immunomodulatory agent (thiopurine or methotrexate).

**Fig. 2:**
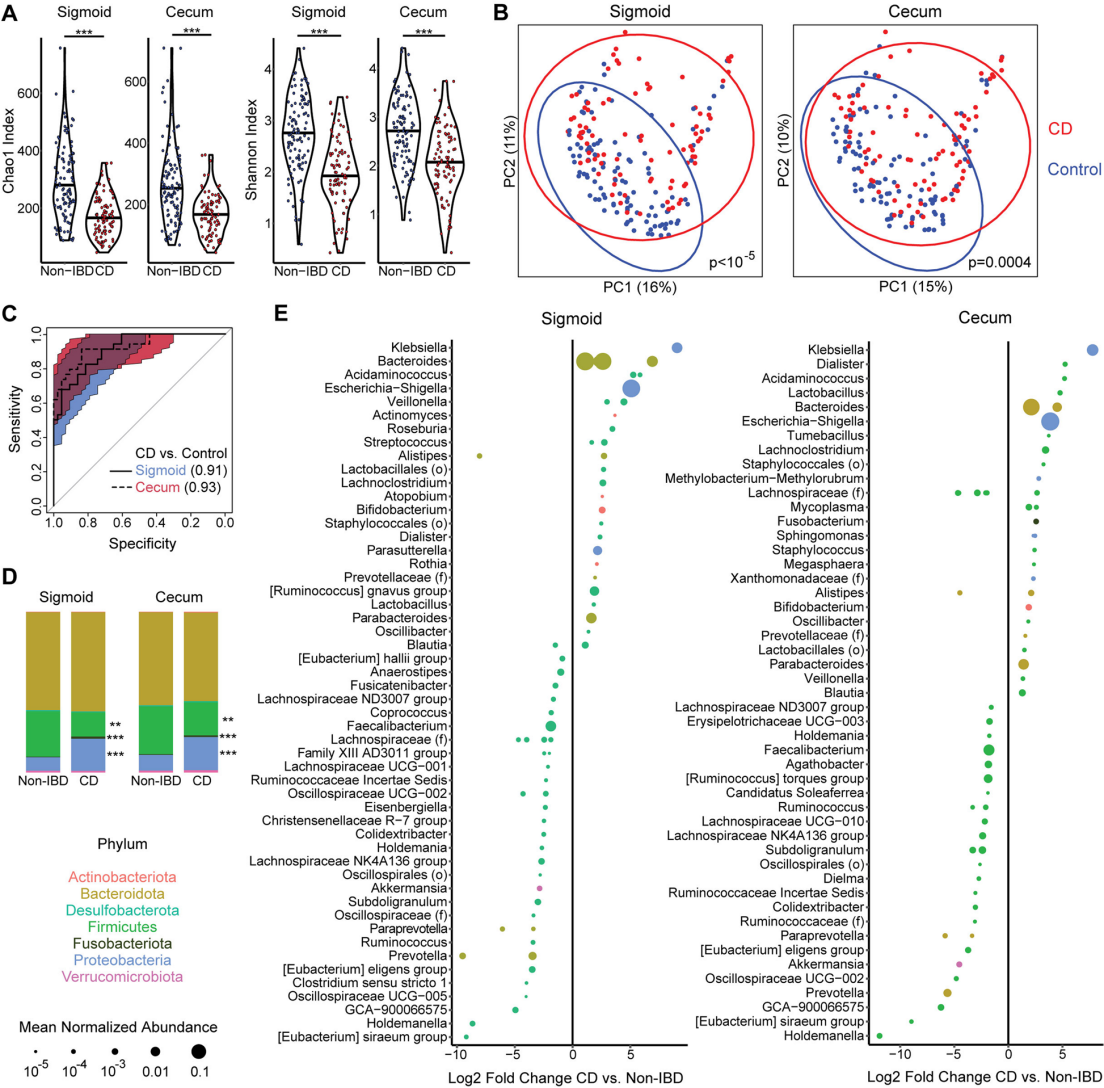
Lower diversity and altered composition of the cecal and sigmoid colon microbiota in CD patients with endoscopically quiescent disease compared to controls. **A** Alpha diversity metrics (Chao1, Shannon) are shown for the cecal and sigmoid MLI microbiome of CD and controls. Significance was determined by ANOVA adjusting for gender, age, and obesity. ***p<10^−4^. **B** PCoA plots based on Bray-Curtis dissimilarity. Color represents IBD status. *p*-values calculated by multivariate PERMANOVA adjusting for gender, age, and obesity. **C** Receiver operating characteristics (ROC) curve for random forest classifiers differentiating CD vs. control subjects based upon cecal and sigmoid MLI microbiota. Area under the curve (AUC) is shown for each site. The colored regions signify the 95% confidence intervals of the curves. **D** Taxonomic summary at the phylum level of the cecal and sigmoid colon MLI microbiome of CD and controls. *q<0.05, **q<0.005, ***q<10^−4^ in DESeq2 models adjusting for gender and obesity. **E** ASVs with a statistically significant difference in relative abundance between CD and controls in multivariate DESeq2 models adjusting for gender and obesity are shown separately for sigmoid and cecum

MLI samples from the cecum and sigmoid colon underwent high-depth 16S rRNA gene sequencing (mean 411,244 sequences per sample) to detect low abundance taxa that may distinguish CD from controls as well as demarcate phenotypic subsets of CD. All analyses were controlled for obesity category (non-overweight, overweight, or obese) given its association with MLI bacterial diversity and composition in controls. The cecal and sigmoid MLI microbiota were found to have lower bacterial diversity in CD patients compared to controls by the Chao1 index of richness (*p*=6 × 10^−9^ for sigmoid, *p*=5 × 10^−8^ for cecum; adjusted for age, gender, and obesity status) and the Shannon index of richness and evenness (*p*=9 × 10^−8^, *p*=1 × 10^−6^ adjusted for age, gender, and obesity) (Fig. 2A). Beta diversity analysis demonstrated a highly significant association of CD status with bacterial composition in both the sigmoid and cecum MLI adjusting for gender, age, and obesity status (Fig. 2B).

To assess whether MLI bacterial profiles could be used to predict CD vs. control status, the cohort was divided into training and test subsets and random forest classifiers were created using the training data. Classifiers based on sigmoid and cecal MLI bacterial profiles had high accuracy to differentiate CD vs. controls when applied to the test data, with area under the curve of 0.91 (95% confidence interval (CI) 0.85–0.96) and 0.93 (95% CI 0.86–0.98), respectively (Fig. 2C). At the phylum level, CD was characterized by expansion in both the sigmoid and cecal MLI of Proteobacteria (*p*=3 × 10^−22^, *p*=4 × 10^−7^) and Fusobacteriota (*p*=3 × 10^−15^, *p*=4 × 10^−6^) with concomitant reduction in Firmicutes (*p*=0.002, *p*=0.009) (Fig. 2D). At the ASV level, CD was associated with enrichment of 31 ASVs in the sigmoid and 31 ASVs in the cecum, with depletion of 52 ASVs in the sigmoid and 41 ASVs in the cecum (Fig. 2E). The most strongly enriched ASV in CD in both the sigmoid and cecum was identified as a member of the *Klebsiella* genus. There was also strong enrichment of a highly abundant ASV identified as belonging to *Escherichia/Shigella* and several abundant ASVs identified as *Bacteroides*. Additional potential pathobionts were enriched including *Fusobacterium*, *Staphylococcus*, *Streptococcus*, *Rothia*, and *Mycoplasma* spp. Conversely, there was depletion of members of *Akkermansia*, *Prevotella*, and many genera within the Firmicutes phylum including *Faecalibacterium*. These taxonomic shifts in the CD MLI microbiome were summarized by CD dysbiosis indices, generated by calculating the log ratio of relative abundances of taxa enriched in CD to abundances of taxa depleted in CD. CD patients had highly significant increases in cecal and sigmoid dysbiosis indices relative to controls with BMI <25 or 25–30 but were not statistically different from obese controls (Fig. 1E). Interestingly, CD dysbiosis index was significantly greater in obese subjects compared to those with BMI<25 in the sigmoid (*p*=0.01) and trended higher in the cecum (*p*=0.07), consistent with taxonomic shifts in obesity paralleling those in CD.

### Biogeographic differences between the cecal and sigmoid colon MLI microbiome are disrupted in CD

**Fig. 3:**
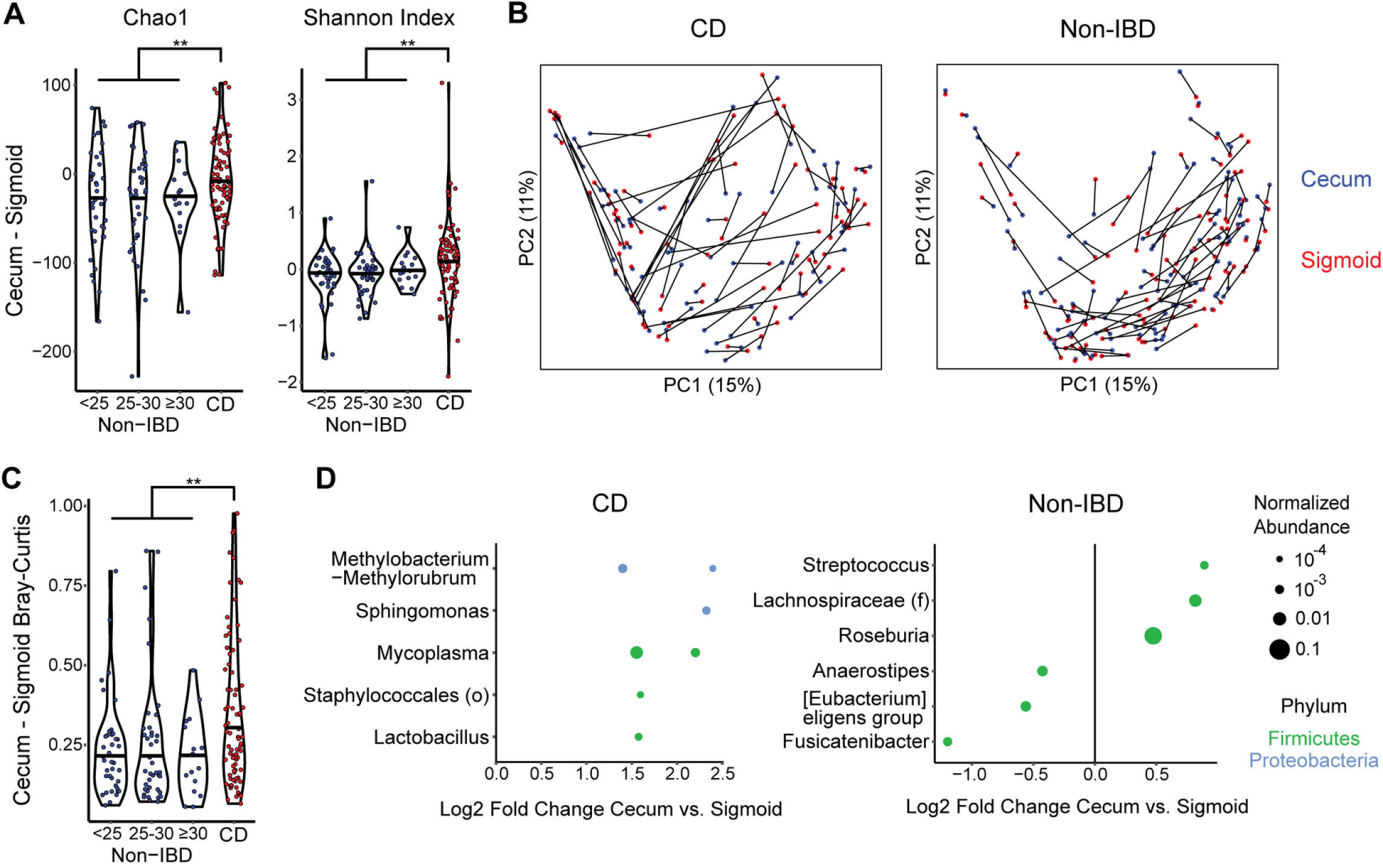
Disruption of the biogeographic relationship between the cecal and sigmoid MLI microbiome in quiescent CD. **A** The per-subject differences in alpha diversity (Chao1, Shannon) between cecum and sigmoid MLI samples are shown for CD and controls divided by BMI category. Significance of differences between CD and non-IBD was determined by the Wilcoxon rank-sum test. **p<0.01. **B** PCoA plots of Bray-Curtis dissimilarity for CD and controls depicting each subject as a line with the ends colored by site (cecum vs. sigmoid). **C** Bray-Curtis dissimilarity between paired cecum and sigmoid MLI samples are shown for CD and controls divided by BMI category. **p<0.01 Wilcoxon rank-sum test. **D** ASVs with a statistically significant difference in relative abundance between cecum and sigmoid in DESeq2 models adjusted for subject are shown for CD and controls

We next investigated what MLI microbial features differentiated the cecum and sigmoid colon and whether these biogeographic differences were affected by CD. First, we evaluated differences in alpha diversity between cecal and sigmoid MLI samples in a paired analysis. The cecum of non-IBD controls had reduced richness compared to the sigmoid by the Chao1 index (*p*=9 × 10^−7^) and a non-significant trend towards reduced Shannon index (*p*=0.08). CD subjects, in contrast, had no significant difference in microbial diversity between the cecum and sigmoid in paired analysis. Moreover, the pairwise differences in cecum vs. sigmoid bacterial diversity of CD subjects were significantly higher than those of non-IBD controls by both the Chao1 (*p*=0.008, −6.5 vs. −28.8) and Shannon indices (*p*=0.006, 0.13 vs. −0.05) (Fig. 3A). This indicates increased relative bacterial diversity in the cecum compared to the sigmoid in CD even though CD was characterized overall by reduced diversity compared to controls. There were no differences across BMI categories within the non-IBD controls. An analysis was then performed of pairwise differences in beta diversity between the cecum and sigmoid colon using Bray-Curtis dissimilarity. Substantial intra-individual variation was seen between cecal and sigmoid bacterial composition, which was significantly greater in CD patients than in controls (*p*=0.005) (Fig. 3B, C). This suggests that Crohn’s disease, even in remission, results in reconfiguration of the biogeographic relationship to not only reverse the alpha diversity gradient seen in controls but also increase the difference in bacterial composition between the cecum and sigmoid colon. In comparison, no difference was seen across BMI categories within the non-IBD controls. In non-IBD controls, 7 ASVs significantly differed between cecum and sigmoid adjusting for subject, including cecal enrichment of *Streptococcus* and *Roseburia* ASVs and depletion of an *Anaerostipes* ASV (Fig. 3D). CD subjects had 6 differential ASVs between sigmoid and cecum that did not overlap with the ASVs seen in non-IBD and included enrichment of *Mycoplasma*, *Sphingomonas*, and *Lactobacillus* spp. in the cecum.

### CD genetic risk is associated with alterations in MLI microbiota

**Fig. 4:**
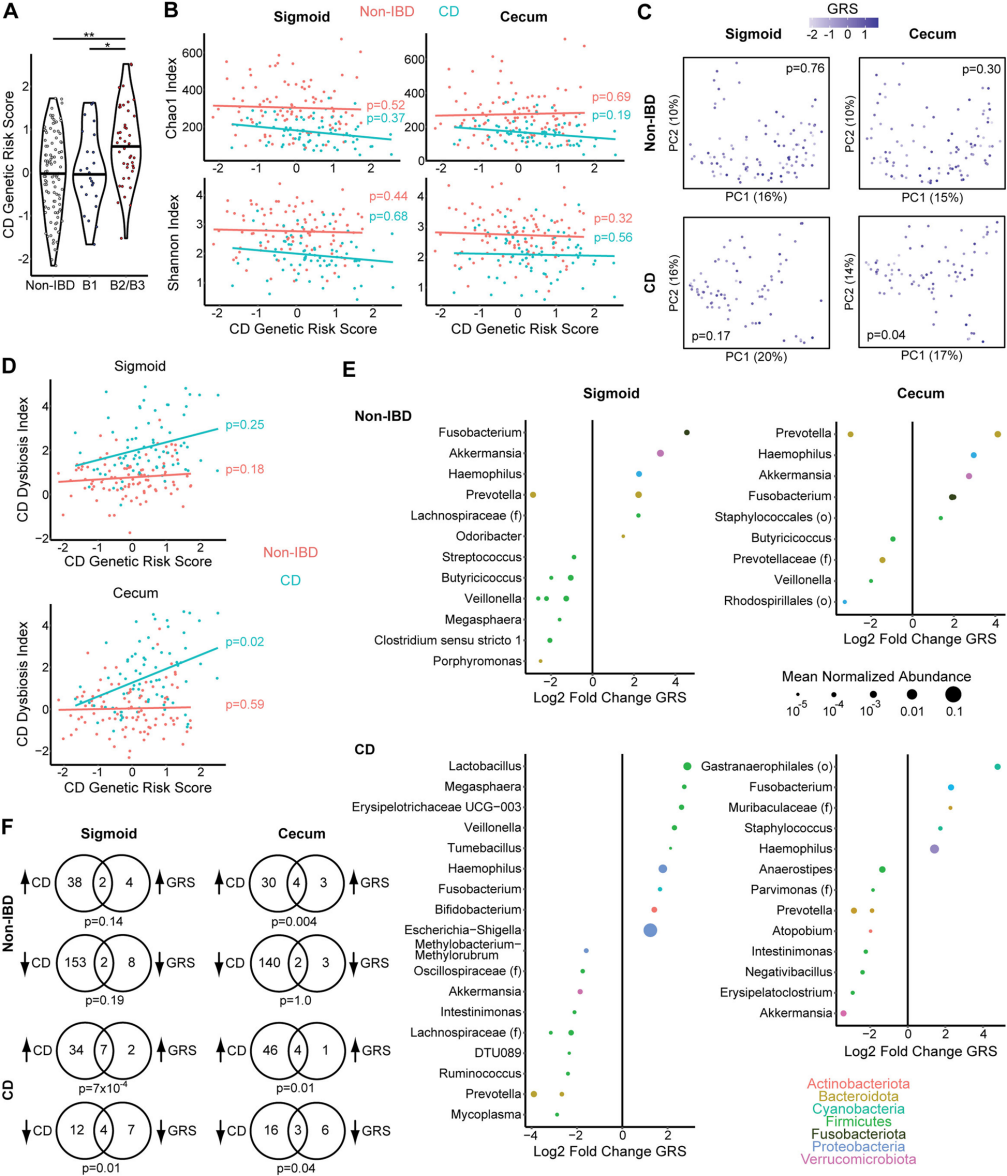
CD genetic risk is associated with taxonomic shifts in the MLI microbiome of CD patients and non-IBD controls. **A** CD genetic risk score for non-IBD controls and CD patients divided by disease behavior (B1 and B2/B3). **B** Alpha diversity metrics are shown by CD genetic risk score for non-IBD controls and CD patients, differentiated by color with separate regression lines. **C** PCoA plots visualizing the cecal and sigmoid microbiome of CD patients, with color scales representing CD genetic risk score (GRS). *p*-values calculated by multivariate PERMANOVA adjusting for gender, age, and obesity. **D** CD dysbiosis index is shown by CD genetic risk score for non-IBD controls and CD patients. Significance in panels **A**, **B**, and **D** was determined by ANOVA adjusted for gender, age, obesity, and disease behavior (for CD patients). **E** ASVs in the cecal and sigmoid MLI microbiome that were significantly associated with CD GRS (*q*<0.1) adjusting for gender, obesity, and CD disease behavior (for CD patients). **F** Venn diagrams depicting overlap of GRS-associated ASVs with ASVs that were enriched or depleted in CD vs. controls in the same region (sigmoid or cecum). Analyses were performed separately in non-IBD controls and CD patients. Significance of overlap was determined by Fisher’s exact test

The inclusion in this cohort of both CD in endoscopic remission and non-IBD controls provided an opportunity to investigate the effects of CD-associated genetic risk factors on the MLI microbiome. As this study was not powered to individually assess the greater than 200 loci genetically linked to CD, we instead used the strategy of summarizing genetic risk in subjects with a polygenic risk score. This score was calculated by summing the log odds ratios from 186 single-nucleotide polymorphisms associated with CD. As anticipated, this genetic risk score (GRS) was significantly higher in the CD subjects than non-IBD controls (Table 1). GRS was also significantly higher in complicated CD than uncomplicated CD; uncomplicated CD and non-IBD controls had equivalent GRS (Fig. 4A). All GRS analyses involving CD subjects were therefore adjusted for disease behavior. There was no significant association of GRS with alpha diversity in non-IBD and CD subjects analyzed separately, adjusting for gender, age, and CD disease behavior (for CD subjects) (Fig. 4B). GRS was significantly associated with cecal but not sigmoid MLI microbial composition in CD subjects (*p*=0.04) when evaluated as a continuous variable in adonis analysis of Bray-Curtis dissimilarity adjusted for gender, age, and CD behavior (Fig. 4C). There was no association of GRS with variation in Bray-Curtis dissimilarity in adonis analyses of non-IBD controls. Similarly, GRS was associated with CD dysbiosis index in the cecum of CD subjects (*p*=0.02) but the trend did not reach significance in the sigmoid and there was no association in non-IBD controls (Fig. 4D). Differential abundance testing demonstrated an association of higher GRS with taxonomic shifts including enrichment of a *Fusobacterium* ASV in the cecum of CD subjects and both the cecum and sigmoid of non-IBD controls, as well as depletion of a *Prevotella* ASV in the sigmoid and cecum of both CD and non-IBD (Fig. 4E). Interestingly, an *Akkermansia* ASV was positively associated with GRS in both the cecum and sigmoid of non-IBD controls but was negatively associated with GRS in the cecum of CD subjects. In CD patients, ASVs associated with higher GRS significantly overlapped with ASVs that were enriched or depleted in CD compared to non-IBD controls in both the cecum and sigmoid (Fig. 4F). In non-IBD controls, ASVs enriched with higher GSR significantly overlapped with ASVs enriched in CD compared to controls in the cecum.

### CD complicated by penetrating or stricturing disease is characterized by increased MLI dysbiosis

**Fig. 5:**
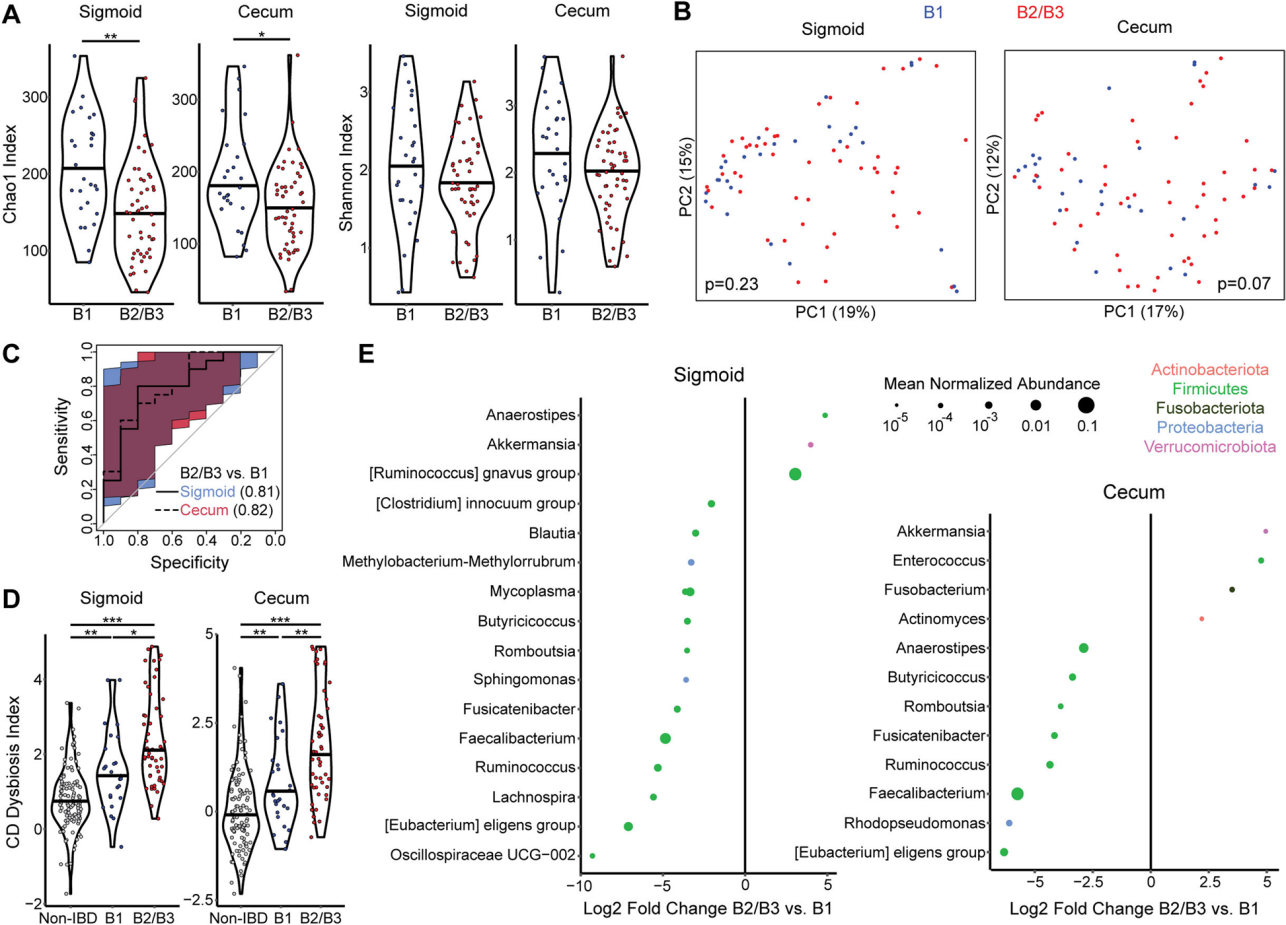
Stricturing/penetrating CD is associated with lower diversity and taxonomic shifts in the MLI microbiome. **A** Alpha diversity metrics (Chao1, Shannon) are shown for the cecal and sigmoid colon MLI microbiome of CD patients stratified by uncomplicated (B1) or complicated (B2/B3) disease behavior according to the Montreal classification. Significance was determined by ANOVA adjusting for gender, age, and obesity. *p<0.05, **p<0.01. **B** PCoA plots based on Bray-Curtis dissimilarity showing the cecal and sigmoid microbiome of CD patients, colored by disease behavior. *p*-values calculated by multivariate PERMANOVA adjusting for gender, age, and obesity. **C** ROC curves for random forest classifiers differentiating CD patients with complicated CD compared to those with uncomplicated CD using MLI bacterial profiles in the sigmoid or cecum. **D** CD dysbiosis index is shown for non-IBD controls and CD patients stratified into B1 and B2/B3. *p<0.05, **p<0.01, ***p<0.001. **E** ASVs in the cecal and sigmoid MLI microbiome that were differentially abundant between complicated and uncomplicated CD controlling for gender and obesity

Having confirmed that CD is characterized by distinct MLI bacterial composition compared to controls during endoscopic remission, we proceeded to investigate whether MLI bacterial composition and diversity were associated with disease behavior. We compared CD patients with complicated disease based upon the Montreal classification (B2=stricturing or B3=internal penetrating) to those with uncomplicated disease (B1). Lower bacterial diversity was seen in the sigmoid and cecum in patients with complicated CD (B2/B3). This reached significance for the Chao1 index (*p*=0.004, *p*=0.04) but not the Shannon index (*p*=0.1, *p*=0.1) after adjusting for gender, age, and obesity (Fig. 5A). Beta diversity analysis using Bray-Curtis dissimilarity did not identify statistically significant differences in bacterial composition by disease behavior adjusting for gender, age, and obesity, though there was a trend towards significance in the cecum (*p*=0.07) (Fig. 5B). Nevertheless, taxa level differences were sufficient to construct random forests classifiers to differentiate complicated (B2 or B3) vs. uncomplicated (B1) disease. Classifiers were trained using data from 60% of CD patients and tested on the remaining 40% of CD patients. In the test subset, sigmoid and cecum MLI classifiers had AUC of 0.81 (95% CI 0.61–0.96) and 0.82 (95% CI 0.63–0.96) (Fig. 5C). Moreover, complicated CD was associated with statistically significant increases in CD dysbiosis index compared to uncomplicated CD in both the sigmoid and cecum (Fig. 5D). Differential abundance testing demonstrated enrichment of *Fusobacterium*, *Enterococcus*, *Actinomyces*, *[Ruminococcus] gnavus* group, and *Akkermansia* ASVs in subjects with complicated CD (Fig. 5E). Conversely, uncomplicated CD was associated with higher relative abundance of many ASVs in genera within the Firmicutes phylum, including those associated with short-chain fatty acid production such as *Faecalibacterium* and *Ruminococcus* that were depleted in CD compared to non-IBD controls (Figs. 2E and 5E).

### MLI microbiome predicts future risk of CD progression

**Fig. 6:**
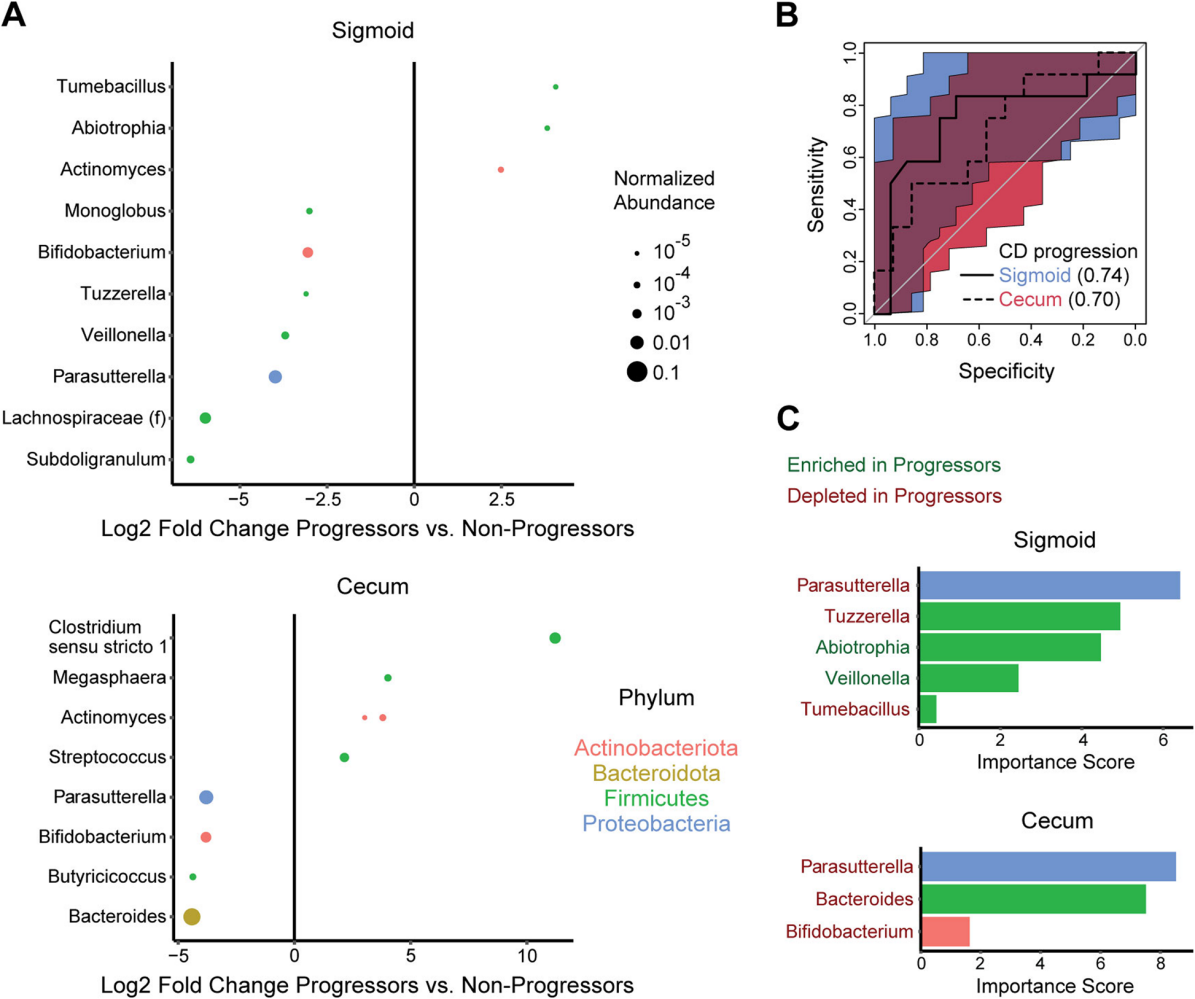
MLI microbiome is associated with risk of Crohn’s disease progression. **A** ASVs in the cecal and sigmoid MLI microbiome significantly associated with disease progression over a mean follow-up period of 3.7 years after adjusting for gender, obesity, and CD disease behavior. **B** ROC curves for random forest classifiers predicting future disease progression from MLI bacterial profiles in the sigmoid or cecum. **C** Importance scores of ASVs included in the sigmoid and cecal random forest classifiers for disease progression. Bar color represents phylum and the color of the genus annotations to the left indicates whether the ASVs were enriched or depleted in CD progressors

Given the association of the MLI microbiome with CD disease behavior, we investigated whether MLI microbial profiles could also predict future CD disease course. Follow-up clinical assessment was available for 72 CD patients, with 32 (44%) showing disease progression after MLI sampling. Progressors were identified by clinician chart review as having any of the following during the period between the index colonoscopy and chart review: hospitalization for CD, episode of bowel obstruction, surgery to treat CD, or requirement to change IBD medication. Follow-up time after MLI sampling was equivalent between progressors and non-progressors (mean 3.63 years vs. 3.76 years, *p*=0.64). There was no significant difference in sigmoid or cecal alpha diversity between CD patients who progressed vs. non-progressors by the Chao1 index (*p*=0.10, *p*=0.33) and the Shannon index (*p*=0.94, *p*=0.73) adjusting for gender, age, obesity, and disease behavior. There were also no significant differences in sigmoid or cecal MLI beta diversity by CD progression status adjusting for gender, age, obesity, and disease behavior (*p*=0.49, *p*=0.58). Sigmoid and cecal CD dysbiosis indices did not significantly differ between progressors and non-progressors adjusting for gender, age, obesity, and disease behavior (*p*=0.99, *p*=1.0). However, progressors had differential relative abundances of multiple ASVs compared to non-responders, including depletion of a *Parasutterella* ASV in both sigmoid and cecum (Fig. 6A). These taxa were used to construct random forests classifiers for CD progression which had AUC of 0.74 (95% CI 0.52–0.93) and 0.70 (95% CI 0.48–0.89) using sigmoid or cecal MLI profiles (Fig. 6B). In both random forest classifiers, the *Parasutterella* ASV made the greatest contribution to classifier accuracy, followed closely in the cecal classifier by a *Bacteroides* ASV that was depleted in progressors (Fig. 6C).

### CD is characterized by shifts in the MLI metabolome including increased cholate to deoxycholate ratio

**Fig. 7:**
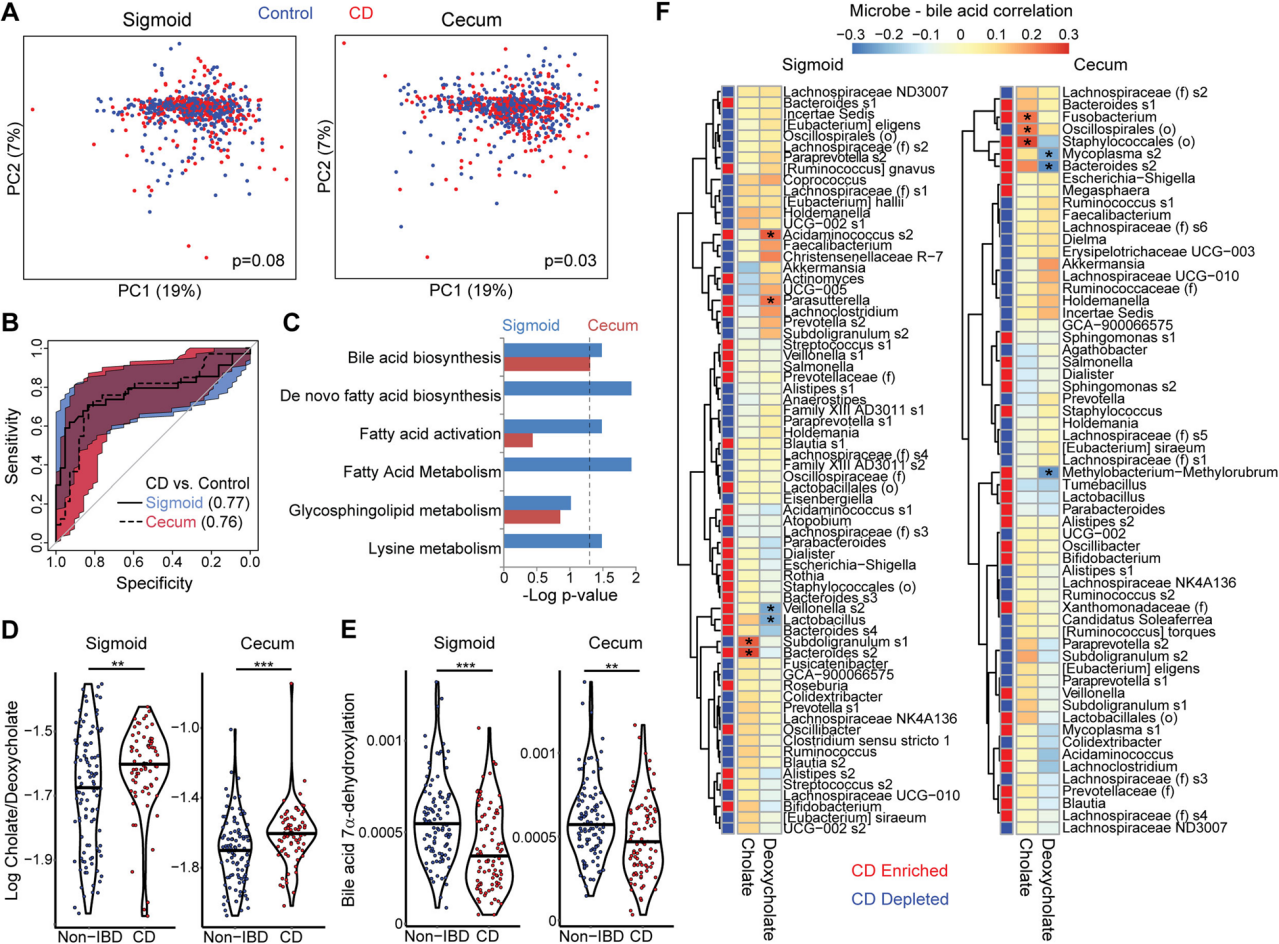
CD is associated with shifts in the metabolome of the cecal and sigmoid MLI. **A** Metabolomics profiles of cecal and sigmoid MLI samples were visualized by PCoA. *p*-values calculated by multivariate PERMANOVA adjusting for gender, age, and obesity. **B** ROC curves for random forests classifiers differentiating CD from controls based on cecal and sigmoid MLI metabolomics features. **C** Metabolic pathways enriched in differential spectral features were identified by mummichog. The negative log of adjusted *p*-values is shown for each pathway; the dashed line represents *p*=0.05. **D** Log ratios of cholate to deoxycholate are shown for non-IBD controls and CD patients. Significance determined by Wilcoxon rank-sum test. **p<0.01, ***p<0.001. **E** Relative abundances of the bile acid 7α-dehydroxylation pathway (conversion of cholate to deoxycholate) in the PICRUSt2 predicted metagenome. Significance determined by Wilcoxon rank-sum test. **p<0.01, ***p<0.001. **F** Heat map and hierarchical clustering showing partial correlations of ASVs enriched or depleted in CD with cholate and deoxycholate, adjusted for disease status, gender, age, and obesity. Red and blue boxes adjacent to the dendogram indicate whether ASVs were enriched or depleted in CD. Significant correlations (*q*<0.05) are indicated by an asterisk

LC-MS untargeted metabolomics analysis was performed on the MLI samples (Additional files 1 and 2, for positive and negative ESI mode metabolomics, respectively). The global MLI metabolomics profiles of CD patients significantly differed from that of controls in the cecum (p=0.03) and trended towards significance in the sigmoid colon (*p*=0.08) (Fig. 7A). Random forests classifiers based on metabolomics data had moderate accuracy for differentiating CD vs controls with AUC of 0.77 (0.65–0.88) and 0.76 (0.62–0.85) for sigmoid and cecum, respectively (Fig. 7B). Mummichog analysis of the list of putatively identified differentially abundant spectral features demonstrated that the following pathways were significantly enriched in the sigmoid: bile acid biosynthesis, de novo fatty acid biosynthesis, fatty acid activation, fatty acid metabolism, and lysine metabolism (Fig. 7C). Of these, only bile acid biosynthesis was also enriched in cecum. Spectral features assigned by mummichog to the bile acid biosynthesis pathway included two with single annotations for cholate and deoxycholate. In both the sigmoid and cecum, CD showed increased cholate (primary bile acid) and decreased deoxycholate (secondary bile acid) relative to non-IBD controls, resulting in highly significant differences in the log ratio of cholate:deoxycholate (Fig. 7D). Correlation analysis was then performed to identify microbes associated with levels of these two bile acids after adjusting for disease status, gender, and obesity. Six significant microbe-bile acid correlations were identified in the sigmoid and six in the cecum (Fig. 7F). Microbes that were enriched in CD and positively associated with cholate included a *Fusobacterium* ASV (cecum), an ASV within the Staphylococcales order (cecum), and a *Bacteroides* ASV (sigmoid). ASVs that were enriched in CD and negatively associated with deoxycholate included *Veillonella* (sigmoid), *Lactobacillus* (sigmoid), *Mycoplasma* (cecum), *Methylobacterium-Methylorubrum*, and the same *Bacteroides* ASV that were positively correlated with cholate (cecum). Given the association of microbes with bile acid levels, we investigated whether CD was associated with a shift in predicted abundance of genes involved in bile acid 7α-dehydroxylation, which mediates bacterial conversion of cholate to deoxycholate. CD was associated with decreased abundance of this pathway in both sigmoid and cecum MLI, consistent with the increased cholate:deoxycholate ratio at both sites (Fig. 7E).

## Discussion

We report the findings of the first moderate- to large-sized study characterizing the colonic mucosal microbiome in Crohn’s disease during endoscopic remission compared to that of non-IBD controls. Similar to prior studies of the fecal and tissue microbiome, CD patients had lower MLI bacterial diversity and altered composition compared to controls [8–15]. One of the major caveats of these past studies has been the potential contribution of active intestinal inflammation to the observed microbial profiles, as animal model studies have demonstrated that induction of intestinal inflammation by itself triggers characteristic shifts in the microbiome including expansion of aerotolerant bacteria and human studies have found that anti-inflammatory therapies reduce dysbiosis [12, 41, 76]. In this study, we detected a robust CD bacterial signature even in the absence of endoscopic inflammation, supporting that bacterial changes are a distinctive feature of CD pathophysiology separate from secondary effects of intestinal inflammation on the microbiome. However, as histological or biochemical parameters of inflammation were not assessed, it remains possible that low-grade inflammation that was not evident endoscopically could contribute to the observed microbiome alterations. Of note, similar results were obtained in a recent study which assessed the microbiome of colonic tissue biopsies taken from 15 non-inflamed CD patients and 16 non-IBD controls [77]. Interestingly, CD not only affected MLI bacterial profiles in the cecum and sigmoid colon considered separately, but also changed the relationship between these two intestinal regions. CD patients showed reduced intra-individual similarity between the sigmoid and cecum as well as increased bacterial diversity in the cecum relative to the sigmoid. While IBD-associated shifts in microbial abundances can vary by colonic region, to our knowledge this is the first study to assess for perturbation of biogeographic relationships across colonic sites [10].

Our study demonstrated altered abundance of a wide range of taxa in the CD MLI microbiome during endoscopic remission compared to that of controls, including marked expansion of *Escherichia-Shigella* and *Klebsiella* spp. This is consistent with prior reports that the Enterobacteriaceae family, which includes *Escherichia-Shigella* and *Klebsiella*, is highly enriched in biopsies from CD patients compared to controls [10–12, 16]. There is an extensive literature demonstrating that adherent-invasive strains of *E. coli* isolated from CD patients can promote colitis and enteritis in animal models [78]. In our study, *Klebsiella* showed an even more prominent enrichment than *Escherichia-Shigella* in CD compared to controls. *Klebsiella* enrichment has been previously reported in the fecal and ileal microbiome of CD patients; this study would be the first to report colonic enrichment [12, 77]. *Klebsiella*, including strains of human-derived oral *Klebsiella pneumoniae*, has been reported to promote animal models of colitis [79, 80]. The strong colonic mucosal *Klebsiella* signature identified here supports the human relevance of these preclinical studies and the possibility that the MLI is an important ecologic niche for oral-derived pathobionts in the gastrointestinal tract. CD was also characterized by expansion of several highly abundant *Bacteroides* ASVs, matching the findings of a large fecal shotgun metagenomics study which reported enrichment of *B. fragilis* and *B. vulgatus* in CD [16]. *B. vulgatus* and enterotoxigenic strains of *B. fragilis* have been shown to promote experimental models of colitis [81, 82]. The MLI CD microbiome was also characterized by global depletion of Firmicutes, which encompassed members of a wide range of genera including *Faecalibacterium*, *Ruminococcus*, *Anaerostipes*, *Coprococcus*, and *Clostridium*, all of which have been previously reported to be depleted in the fecal and/or colonic tissue microbiome of CD patients [12, 14, 34]. Of these, the most consistent association in the literature has been depletion of *Faecalibacterium prausnitzii*, which has been shown to reduce severity of experimental colitis through production of anti-inflammatory products such as butyrate and a novel protein, MAM [83, 84]. Collectively, bacterial taxa that were enriched or depleted in CD were used to construct a CD dysbiosis index which could be used to summarize MLI bacterial shifts associated with CD.

This study also provided insight into the mucosal microbiome of obesity. Despite the compelling preclinical data supporting a role for the microbiome in obesity, specific taxonomic signatures of human obesity have been elusive due to variability across fecal microbiome studies [85–90]. The potential importance of the mucosal microbiota in obesity is supported by reports that obese individuals have increased intestinal permeability and translocation of gut microbes into the mesenteric adipose tissue [91, 92]. Strikingly, we found that obese non-IBD controls had similar MLI bacterial characteristics as CD, including reduced diversity, expansion of pathobionts such as *Escherischia-Shigella*, and depletion of beneficial microbes such as *Faecalibacterium*. This was reflected as a significant elevation in CD dysbiosis index compared to controls with BMI <25. Among the obesity-associated shifts in the MLI microbiome, enrichment of *Escherichia-Shigella* and *Megasphera* and depletion of *Faecalibacterium*, *Paraprevotella*, and *Methanobrevibacter* have been reported in at least one prior fecal microbiome study [93]. These findings indicate that mucosal dysbiosis is a common thread between CD and obesity—both diseases rising rapidly in incidence in the Western world [85]. This is consistent with the current understanding of obesity as a low-grade inflammatory state and suggests that mucosal dysbiosis could contribute to inflammation in the settings of both obesity and IBD.

Complicated CD with either stricturing or penetrating disease behavior was associated with reduced MLI microbial diversity and increased CD dysbiosis index relative to uncomplicated CD. This is consistent with a recent study of colon tissue biopsies from 15 CD patients which observed reduced diversity in complicated CD (B2 and B3) compared to uncomplicated CD [77]. There is little existing literature on specific colonic mucosal taxa that distinguish CD disease behaviors. One study of newly diagnosed pediatric CD patients reported that those who went on to progress to B2 or B3 had increased levels of *Ruminococcus* and *Collinsella* and reduced levels of *Rothia* and *Veillonella* in rectal tissue biopsies obtained at initial presentation [34]. These taxa do not overlap with those identified in this study, potentially reflecting the effects of active inflammation in the prior study as well as differences in the microbes that are associated with future disease behavior as opposed to established disease behavior. In this study, *Akkermansia* enrichment and *Faecalibacterium* depletion were observed in both the sigmoid and cecum of complicated CD. The association of reduced *Faecalibacterium* with CD complications supports the concept that the anti-inflammatory effects of these microbes protect against development of disease complications. Relative expansion of *Akkermansia* sp. in complicated CD was surprising as this microbe was reduced overall in CD compared to controls. This suggests a complex role for *Akkermansia* spp. in CD, consistent with preclinical studies which have found that *Akkermansia* can reduce or exacerbate intestinal inflammation depending on the animal model [94, 95]. Significant enrichment of a *Fusobacterium* ASV in complicated CD was seen in the cecum but not the sigmoid. This suggests that *Fusobacterium*—oral pathobionts implicated in colorectal cancer that can also exacerbate intestinal inflammation—may contribute to disease processes in the proximal colon that promote stricturing and/or penetrating disease [96, 97].

MLI microbial profiles were also found to predict future CD disease progression. To date, much of the literature on microbial biomarkers of CD disease course has centered on the risk of post-operative reoccurrence after ileal or ileocecal resection. These studies have demonstrated that ileal mucosal microbial profiles at the time of resection are highly predictive of recurrence whereas fecal microbial profiles have comparatively weak accuracy [83, 98, 99]. One study of longitudinally collected fecal samples from a cohort of 45 CD patients in clinical remission followed for 2 years identified 17 differential taxa between the 12 patients who flared and those who did not, including *Sutterella*, S24.7, Gemellaceae, and Christensenellaceae [20]. In our study with 72 CD patients in endoscopic remission, the most consistent signals were depletion of *Parasutterella* and *Bifidobacterium* spp. in those who had disease progression. The signature taxa in this study differed from that of the prior study likely due to the use of mucosal rather than fecal samples, differences between cross-sectional and longitudinal assessment, and exclusion of patients in clinical remission with endoscopic signs of inflammation in our study. Among the microbes associated with disease progression in this study, *Parasutterella* made the strongest contribution to classifier accuracy. Interestingly, *Parasutterella* spp. have been previously reported to be greatly enriched in the ileal submucosa of CD patients, suggesting that these microbes have invasive properties [100]. This would be the first report of expansion of these microbes in the CD colon and, if validated, warrants investigation into mechanisms by which these and other mucosal pathobionts can modulate disease course in CD.

Our study also contributes new insights into genetic factors that influence the intestinal microbiome. Existing gene-microbe association studies have largely focused on the fecal microbiome of large healthy cohorts, and analyzed for association of taxa enriched with individual locus variants at genome-wide significance [26, 29, 101–104]. However, it is notable that while host traits reflect the combinatorial effect of genetic loci, few studies have evaluated the relationship of summary measures of IBD genetic risk with the microbiome. We are aware of only one such study that reported an association of a genetic risk score derived from 11 loci with reduced fecal *Roseburia* in healthy controls but not IBD patients [105]. In the present study, we found that a comprehensive genetic risk score comprising 186 known variants was significantly associated with microbial composition and higher CD dysbiosis index in the cecum of CD patients. This relationship did not reach significance in the sigmoid, suggesting greater impact of genetic burden on the microbiome in the proximal colon. A strength of this study is the assessment of a summary metric of IBD genetic risk on microbiome composition in non-IBD subjects, which isolates their microbiome impact from that related to disease state. Controls showed taxonomic changes with higher GRS including enrichment of *Fusobacterium* but did not show an association of GRS with CD dysbiosis index. This suggests that in the absence of disease, only specific mucosal taxa are responsive to global genetic risk for CD and that genetic modulation of mucosal responses in CD may contribute to the association of GRS with dysbiosis in CD.

MLI sampling also offered insight into metabolite shifts in the colonic mucosa of CD patients. In particular, we observed an increased ratio of a primary bile acid, cholate, relative to its corresponding secondary bile acid, deoxycholate. These findings suggest a reduction in bacterial transformation of primary to secondary bile acids and are consistent with a recent fecal metabolomics study reporting increased cholate and decreased deoxycholate in CD patients with dysbiosis as well as findings from preclinical models of intestinal injury [19, 106]. The shift towards greater primary bile acid in the CD MLI was associated with alterations of specific MLI taxa which could reflect either a contribution of these microbes to bile acid transformation—consistent with reduced predicted abundance of genes in the bile acid 7α-dehydroxylation pathway—or downstream effects of altered bile acid metabolism on these microbes. The latter possibility is consistent with the known wide-ranging effects of bile acid receptors such as FXR on intestinal physiology including production of antimicrobial products [107]. Of note, these correlations were based on microbial relative abundances and different relationships may be observed between metabolite levels and absolute abundances of mucosal bacteria. These findings highlight a potential role for altered bile acid metabolism in mucosal dysbiosis in IBD and demonstrate that metabolomics analysis of MLI samples can be used to characterize mucosal host-microbe interactions in diseases such as CD.

Beyond the insights that can be gained from taxonomic and metabolite associations with CD disease phenotypes, this study also suggests the potential clinical application of mucosal microbial profiles as biomarkers for Crohn’s disease. The largest published CD study using colonic mucosal samples reported a classifier for distinguishing CD from controls that had AUC 0.78 [11]. The current study builds on this existing literature by demonstrating that colonic MLI microbial biomarkers not only robustly separate CD from controls (AUC 0.91, 0.93), but also distinguish CD disease behaviors with high accuracy even during endoscopic remission (AUC 0.81, 0.82) and can predict future disease progression (AUC 0.74, 0.70). In clinical practice, such biomarker panels could be used to identify CD patients in remission who are at higher risk for flare or disease complications. This could guide clinical decisions on therapies and disease monitoring, a challenge in CD as active disease may not manifest in clinical symptoms until substantial disease progression has occurred. While this study provides significant insights into mucosal microbial markers of CD disease phenotype, it has limitations which include the demographic differences between controls and CD patients, lack of longitudinal sampling, moderate sample size of disease subgroups such as complicated CD, and absence of an independent validation cohort to assess classifier accuracy. Future studies are warranted to address the clinical potential of MLI-based biomarkers for CD clinical management and to investigate individual MLI microbes that may be instigators of inflammation and disease progression.

## Conclusions

During endoscopic remission, Crohn’s disease patients show robust differences in colonic mucosal microbiome diversity, composition, and biogeography compared to unaffected controls. This indicates that microbial changes are a distinctive feature of CD pathophysiology separate from secondary effects of intestinal inflammation on the microbiome. Elements of this mucosal microbiome signature also predict CD behavior and future disease progression, representing potential biomarkers for prognostication and interception therapies. Specific taxa in both CD patients and non-IBD controls were associated with CD genetic risk, providing evidence that pre-disease risk may be mediated by selective microbial shifts in the mucosa. Obese controls showed changes in colonic mucosal microbial composition that paralleled those of CD, suggesting a shared microbial contribution to these two diseases that are both rising in incidence in the Western world.

## Supplementary Information

**Additional file 1.** Positive ESI mode metabolomics: This file contains the positive ESI mode metabolomics data. Each spectral feature is labeled by its m/z and retention time.

**Additional file 2.** Negative ESI mode metabolomics: This file contains the negative ESI mode metabolomics data. Each spectral feature is labeled by its m/z and retention time.

## Data Availability

The sequencing data supporting the conclusions of this article are available in the NCBI Bioproject repository, PRJNA737297 (https://www.ncbi.nlm.nih.gov/bioproject/737297) [108]. The metabolomics data have been deposited in the Metabolomics Workbench repository under ID 3353 (https://www.metabolomicsworkbench.org) and are included as Additional files 1 and 2, representing positive and negative ESI mode metabolomics, respectively [109]. The R code used for bioinformatics analyses are provided in a Github repository (https://github.com/jjgithub650/Mucosal-luminal-interface-microbiome-in-Crohns-disease) [110].

## Abbreviations

ASV: Amplicon sequence variant
AUC: Area under the curve
BMI: Body mass index
CD: Crohn’s disease
CI: Confidence interval
GRS: Gene risk scores
HMDB: Human Metabolome Database
IBD: Inflammatory bowel disease
IGR: Interquartile range
KEGG: Kyoto Encyclopedia of Genes and Genomes
MLI: Mucosal-luminal interface
PCoA: Principal coordinate analysis
ppm: Parts per million
SNP: Single-nucleotide polymorphism
